# Evaluation of Copper Concentration in Subclinical Cases of White Muscle Disease and Its Relationship with Cardiac Troponin I

**DOI:** 10.1371/journal.pone.0056163

**Published:** 2013-02-08

**Authors:** Forough Ataollahi, Mehrdad Mohri, Hesam A. Seifi, Belinda Pingguan-Murphy, Wan Abu Bakar Wan Abas, Noor Azuan Abu Osman

**Affiliations:** 1 Department of Clinical Sciences, School of Veterinary Medicine, Ferdowsi University of Mashhad, Mashhad, Iran; 2 Department of Clinical Sciences and Centre of Excellence in Ruminant Abortion and Neonatal Mortality, School of Veterinary Medicine, Ferdowsi University of Mashhad, Mashhad, Iran; 3 Department of Biomedical Engineering, Faculty of Engineering, University of Malaya, Kuala Lumpur, Malaysia; University of Nebraska Medical Center, United States of America

## Abstract

The present study aims to evaluate the serum level of copper (Cu) in lambs suffering from subclinical forms of white muscle disease (WMD) and its relationship with cardiac troponin I (cTn-I) as a novel biomarker of cardiovascular disorders. Ten milliliters of jugular blood were taken from 200 lambs less than one year old to measure serum concentrations of Cu, selenium (Se), and cTn-I. The subjects were divided into 2 groups, namely, the deficient group which included 36 lambs, and the control group which included 164 lambs according to the reference serum Se concentration (50 ng/mL). Serum Se levels in the deficient group were lower than 50 ng/mL. By contrast, the control group showed Se levels higher than 50 ng/mL. Differences among the serum Cu and cTn-I levels were determined in both groups. The mean ±SD and median of serum Cu and cTn-I levels in the deficient group were lower and higher than those in the control group, respectively. A significant positive correlation was observed between serum Cu and Se levels, and also serum Cu and Se levels showed a negative correlation with serum cTn-I concentrations. Stepwise linear regression analysis showed that serum Cu levels were correlated positively with serum Se levels (p<0.05). Receiver operating characteristic (ROC) curve analysis indicated that the area under curve (AUC) of Cu was significantly higher than that of cTn-I based on the reference diagonal line. It is important to keep in mind that the value of AUC for the ROC curve is between 0.5 and 1.00, in which the lowest accuracy is related to the reference diagonal line with AUC of 0.5. A cut-off was determined to indicate which Cu level can discriminate between affected and healthy lambs. The cut-off level, sensitivity, and specificity of Cu in this study were 144.5 ng/mL, 74%, and 61%, respectively.

## Introduction

Copper (Cu) is an essential trace element that participates in the pathogenesis of numerous heart diseases [1]. This trace element is involved in the structure of antioxidant enzymes such as superoxide dismutase (SOD) in the form of selenoprotein [2], [3], [4]. Activity of this selenoprotein protects cardiac myocyte membranes in the myocardium against oxidative stress-mediated destruction [1]. Moreover, Cu has a catalytic effect on oxidative stress removal through its role in catalase activity [5]. The simultaneous presence of Cu in the structure of SOD and catalase indicates the critical role of Cu in removing oxidative stress [6]. As such, any significant change in the status of serum Cu could lead to alterations in antioxidant enzyme activities and subsequently affect tissue susceptibility to oxidative stress [2], [7], [8] involved in the pathogenesis of white muscle disease (WMD) [9].

Deficiencies in selenium (Se) and vitamin E are defined as main characteristics of the etiology of WMD [10]. Se is a biochemical component of the active site of antioxidant enzymes such as glutathione peroxidase (GSH-PX), which protects the membrane integrity of cardiomyocytes against oxidative stress [1]. Based on the dependence of GSH-PX on the Se supply, a lack of sufficient GSH-PX in WMD induces lipoperoxidation in tissues and, eventually, degeneration and necrosis of the myocardium. Hyaline degeneration and myocardial necrosis could lead to the release of cardiac biomarkers, such as cardiac troponin I (cTn-I) into the blood circulation and finally sudden death [9].

Biochemical markers such as cTn-I [11], [12] can be used to diagnose clinical and subclinical forms of WMD, since cTn-I concentrations increase during the early stages of WMD-mediated myocarditis [10], [13]. An elevated level of cTn-I indicates a greater severity of disease because the serum cTn-I concentration is dependent on the size of ischemic tissue [5]. Although the relationship between Se and cTn-I has already been investigated [9], [10], [14], changes in serum Cu levels in subclinical forms of WMD have yet to be explored in the field of sheep medicine. The main outcome of the current study is the evaluation of serum Cu levels in lambs suffering from subclinical forms of WMD and determination of the correlation between serum Cu and serum cTn-I levels to indicate the severity of disease.

## Materials and Methods

Ethical statement: This study was approved by the ethics committee of Ferdowsi University of Mashhad, Iran, to allow the collection of blood samples from the jugular veins of sheep. To obtain samples, the sacrifice of animals was neither necessary, nor performed.

This study was performed on 200 lambs under one year of age and from different geographical regions and herds of the Khorasan Razavi province in northeastern Iran. These regions and herds have a history of Se deficiency in the soil and hay as well as a history of WMD cases that had been previously referred to the veterinary teaching hospital of the veterinary medicine school in Ferdowsi University of Mashhad, Iran. A total of 186 blood samples were collected from 9 herds. The numbers of samples per herd depended on the size of the herd. Other samples were selected from lambs referred to our teaching hospital. Aside from 6 lambs with the clinical form of WMD (based on clinical signs, necropsy, pathology, and serum Se concentration), some of the lambs suffered from other diseases, including 4 lambs with Peste des Petits Ruminants, 1 lamb with Cu deficiency, 2 lambs with keratitis, 3 lambs with foot-and-mouth disease, 13 lambs with laminitis, and 2 lambs with a respiratory disease.

Jugular blood samples (10 mL) from each lamb were taken using a plain tube for serum harvesting. Blood samples were kept on ice during transfer to the lab. Serum harvesting was achieved by centrifugation at 1800 *g* for 10 min, and serum samples were kept at −20°C until analysis. The Se concentration in serum samples was measured by atomic absorption spectrophotometry (Perkin Elmer 3030, USA). Control serum (Randox control sera, Antrim, UK) was used for controlling measurement accuracy. cTn-I was measured in serum samples using ELISA kits (Monobind Inc., Lake Forest, California, USA), and the results were evaluated according to the manufacturer's instructions. Cu concentration was measured using commercial kits (Randox, Antrim, UK) with an autoanalyzer (Biotechnical, Targa 3000, Rome, Italy). Samples were divided into two groups, a Se-deficient group and a normal group, according to the base concentration of serum Se (50 ng/mL in lambs [15]. The Se-deficient group consisted of 36 lambs (Se<50 ng/mL), and the normal group consisted of 164 lambs (Se>50 ng/mL).

*Statistical analysis* was conducted using SPSS software (Version 16, SPSS Inc., Chicago, USA). Spearman's method was also applied to detect the strength and direction of correlation between the measured parameters of the two groups. The linear association between the independent variable (Cu) and dependent (Se) variable in lambs suffering from WMD was further explored through stepwise linear regression. To evaluate whether or not Cu concentration has prognostic ability in WMD, a receiver operative characteristics (ROC) curve was applied. p≤0.05 was considered significant in all comparisons.

## Results

The mean (SD) and median of serum Cu and serum cTn-I concentrations in the Se-deficient and control groups are shown in Table 1. Serum Cu levels in the deficient group were lower than those in the normal group (p<0.05), while the concentrations of cTn-I were significantly higher in the deficient group than in the control group (p<0.05). Spearman's analysis was used to investigate the relationship between parameters. A positive correlation between serum Cu and serum Se levels was observed, r = +0.26, n = 200, p<0.005. By contrast, serum Cu and serum Se levels showed significant negative correlations with serum cTn-I concentrations, r = −0.37, n = 200, p<0.005 and r = −0.23, n = 200, p<0.005, respectively.

**Table 1:** Comparison of serum Cu level and serum cTn-I levels between deficient and normal Se level groups.

category		Cu (µg/dl)	cTn-I (ng/dl)	values	P_Value_
Deficient	Median	141	0.05	36	0.001
	Mean (SD)	135.800 (27.68)	0.1338 (0.22)		
Normal	Median	154	0.000	164	0.004
	Mean (SD)	155.790(32.92)	0.061 (0.14)		

Deficient group: lambs with serum Se level less than 50 ng/mL.

Normal group: lambs with serum Se level higher than 50 ng/mL.

**Figure 1:**
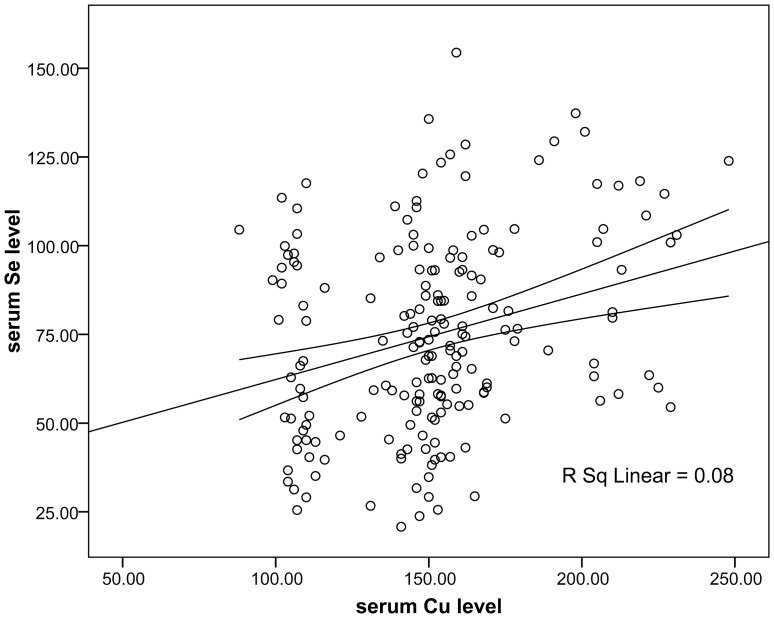
Positive correlation between serum Cu level (independent variable) and serum Se level (dependent variable) in lambs with WMD.

Linear regression as a statistical procedure was used to predict the value of the continuous dependent variable (Se) based on the value of the independent variable (Cu). In this study, stepwise linear regression indicated that serum Cu levels were positively associated with serum Se concentrations (y = 0.24 x+38.8, p<0.001, R^2^ = 0.08, effect size = 0.08) (Fig 1, Table 2).

**Table 2:** Significant contribution of serum Cu levels in changes of serum Se level through the stepwise linear regression.

	P_value_	Correlation coefficient	Values	“R square”
Serum Se levels	0.001	0.282	185	0.08

Predictor: serum Cu level.

Dependent variables: serum Se level, serum cTn-I level.

**Figure 2:**
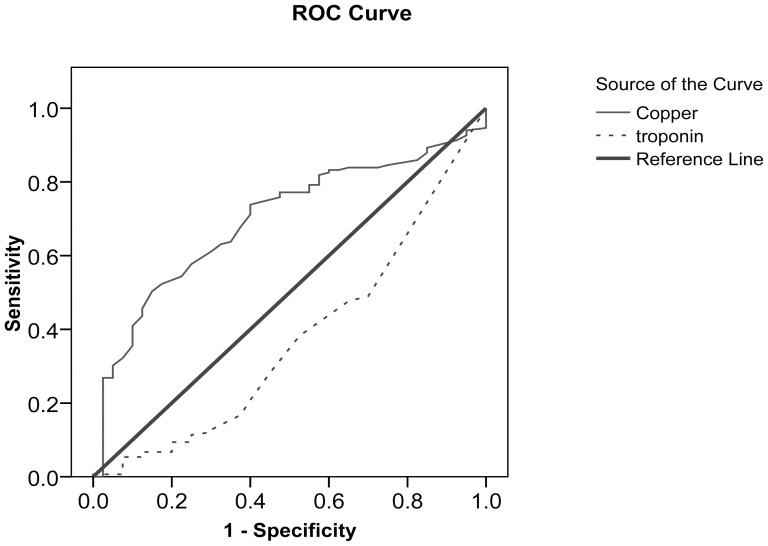
Prognostic ability of serum Cu level regarding to WMD occurrence.

The complete curve of ROC relating serum Cu and serum cTn-I levels presented an overall picture of the diagnostic performance of these parameters (Fig. 2, Table 3). In the comparison of different tests, the efficient curve lean toward the top left corner, whereas the worse case is the reference diagonal line. Hence, the detective indicator in the diagnostic performance of parameters is the area under curve (AUC) of the ROC curve. The diagnostic performance (AUC) of the diagonal reference line, also called the chance line, is 0.5, so curves nearer to the diagonal reference line have poorer diagnostic ability than curves farther from this line [16], [17]. In this study, the applied ROC showed that serum Cu levels have high performance in predicting WMD occurrence, since the AUC of serum Cu levels was greater than that of cTn-I. A proper threshold (cut-off point) for serum Cu levels to indicate which Cu level can discriminate between affected and healthy lambs is 144.5 µg/dL in this study. Furthermore, any point on the ROC curve has specific sensitivity (true positive rate) and specificity (false positive rate). The calculated sensitivity and specificity to determine the usefulness of serum Cu levels in the prediction of WMD were 74%, and 61%, respectively.

**Table 3:** Area under Curve (AUC) – diagnostic performance of serum Cu level and serum cTn-I level according to AUC.

Test Results Variable (s)	Area	Std. Error	Asymptotic Sig.	Asymptotic 95% Confidence Interval	
			`	Lower Bound	Upper Bound
Cu	0.720	0.039	0.000	0.204	0.356
Tn	0.670	0.050	0.001	0.573	0.768

## Discussion

Cu has a protective impact on cell membranes against hydrogen peroxide and superoxide anion involved in pathogens of cardiovascular disorders by SOD as antioxidant enzymes [18]. These free radicals induce hyaline degeneration and myocardial necrosis through lipoperoxidation [9]. Inadequate levels of Cu decrease the activity of antioxidative enzymes against oxidative injury [19] and promote the progress of cardiovascular diseases [18]. This study evaluates serum Cu levels in subclinical forms of WMD and elucidates the relationship between serum Cu levels and serum cTn-I concentration. Serum Cu and serum cTn-I levels were measured in lambs suffering from WMD. The results obtained, based on the median and mean (SD), showed a significant decrease in serum Cu levels in the Se-deficient group in comparison with the normal group. A positive correlation between serum Cu levels and serum Se levels was also observed according to Spearman's test. These findings reveal the mobilization of circulating Cu into the ischemic myocardium during the early stages of WMD and its utilization in the antioxidant system. In other words, the decrease in serum Cu levels in the Se-deficient group may be due to a myocardial need to utilize large amounts of Cu for the repair of cardiac degeneration in subclinical forms of WMD. The findings are in agreement with the study of Sahin et al. (2009) [20], who found that serum Cu levels were significantly lower in beef cattle after transportation because of the involvement of Cu in the antioxidant system. Some studies, however, show high levels of Cu in cardiovascular diseases, contrasting with results of this study [1], [5], [18], [20], [21]. The increased levels of Cu in these opposing studies could be a consequence of Cu release by myocardial necrosis.

Comparisons between the median and mean (SD) of the two groups showed higher levels of cTn-I in the deficient group compared with those in the control group, which could be taken as evidence of myocardial damage in lambs suffering from subclinical forms of WMD. The negative relationship between serum Cu and serum cTn-I levels in Spearman's test illustrates that cardiac injury is an expanding process. Some investigators [9], [10], [14] have reported elevations in cTn-I in WMD, agreeing with our results. Insufficient antioxidant defense in WMD leads to the release of cTn-I into the circulatory system from the cytosolic pool during the first steps of cardiac injury, while extension of damage leads to the release of structurally bound troponin into the blood circulation [9], [22]. The results of stepwise linear regression indicate that serum Cu levels are potentially associated with serum Se levels. Our findings suggest that determination of serum Cu levels has possible clinical utility in the prediction of subclinical stages of WMD in lambs.

A comparison of AUCs of serum Cu and serum cTn-I levels indicates the significantly improved diagnostic ability of serum Cu levels over serum cTn-I levels. The proper cut-off point for serum Cu levels was 144.5 µg/dL. Beyond this serum concentration, lambs with WMD could be discriminated correctly from healthy lambs. In other the words, serum Cu levels <144.5 µg/dL could indicate the presence of WMD and cardiac damage in lambs. The calculated sensitivity and specificity of serum Cu levels for the prediction of WMD were 74% and 61%, respectively. As such, serum Cu levels are useful for predicting the onset of the disease.

Comparisons between this study and other studies show that changes in serum Cu status are associated with the severity of WMD. The increasing size of ischemic tissue leads to the release of higher levels of serum Cu and cTn-I into the blood circulation. Low levels of Cu in the deficient group could indicate no structural damage in the myocardium at early stages of the disease. Indeed, high levels of cTn-I in the deficient group are most likely evidence of early troponin release from the cytosolic pool, the release of which is due primarily to the declined integrity of the cell membrane.

## Conclusion

Depletion in serum Cu levels are a reliable index for diagnosing subclinical stages of WMD and is able to indicate heart involvement at this stage. Early diagnosis using serum Cu levels could prevent poorer outcomes of WMD in herds. Cu chelating, Se administration, and vitamin E injection may be effective therapies against WMD.
